# The psychological impact on an orthopaedic outpatient setting in the early phase of the COVID-19 pandemic: a cross-sectional study

**DOI:** 10.1186/s13018-020-01862-9

**Published:** 2020-08-12

**Authors:** Khai Cheong Wong, Xinyun Audrey Han, Kae Sian Tay, Suang Bee Koh, Tet Sen Howe

**Affiliations:** Department of Orthopaedic Surgery, Singapore General Hospital, Singapore, Singapore

**Keywords:** COVID-19, Mental health, Healthcare workers, Stress, Psychological

## Abstract

**Background:**

World Health Organization declared coronavirus disease-19 (COVID-19) a global pandemic on 11 March 2020, after the coronavirus claimed 4628 lives worldwide. Mental health challenges such as making impossible decisions and working under extreme pressures are expected to be faced by frontline healthcare workers who are directly involved in the care of COVID-19 patients. However, we question if significant stress levels might also be observed in a subspecialty musculoskeletal outpatient department, where staff are not first-line care providers of COVID-19 patients. We hypothesize that these healthcare workers also face significant psychological strain, and we aim to objectively determine the prevalence using a validated caregiver strain index.

**Methods:**

A cross-sectional study was conducted in outpatient musculoskeletal clinics in a tertiary hospital in Singapore. We collected basic demographic data and used a 13-question tool adapted from the validated Caregiver Strain Index (CSI) to measure psychological strain in these healthcare workers. Participants were divided into 2 groups depending on the level of strain experienced.

**Results:**

A total of 62 healthcare workers volunteered for this study. There were 32 participants (51.6%) who had 7 or more positive responses (group 1) and the remaining 30 participants (48.4%) were allocated to group 2. There were no significant differences between the two groups in terms of demographic data. “Work adjustments” (74.2%), “changes in personal plans” (72.6%), and finding it “confining” (72.6%) garnered the most positive responses in the questionnaire. On the other hand, “financial concerns” garnered the least positive responses (21.0%).

**Conclusion:**

The protracted duration of the COVID-19 outbreak and its resultant prolonged adjustments can have unintended consequences of wearing down healthcare resources otherwise allocated to chronic and elective conditions. Countries should ensure that measures are put in place to safeguard the mental well-being of our healthcare workers to avoid needing another reactive strategy in this battle against COVID-19.

## Introduction

The World Health Organization declared coronavirus disease-19 (COVID-19) a global pandemic on 11 March 2020, after the coronavirus claimed 4628 lives worldwide. Literature has been published regarding epidemiology and clinical features of COVID-19, with emerging studies examining the impact on the psyche of frontline healthcare workers. Mental health challenges such as making impossible decisions and working under extreme pressures are expected to be faced by frontline healthcare workers who are directly involved in the care of COVID-19 patients [1]. This group of frontline medical staff was also worried about bringing the virus to their homes, feeling incapable when facing critically ill patients, and shortage of protective equipment [2].

**Fig. 1:**
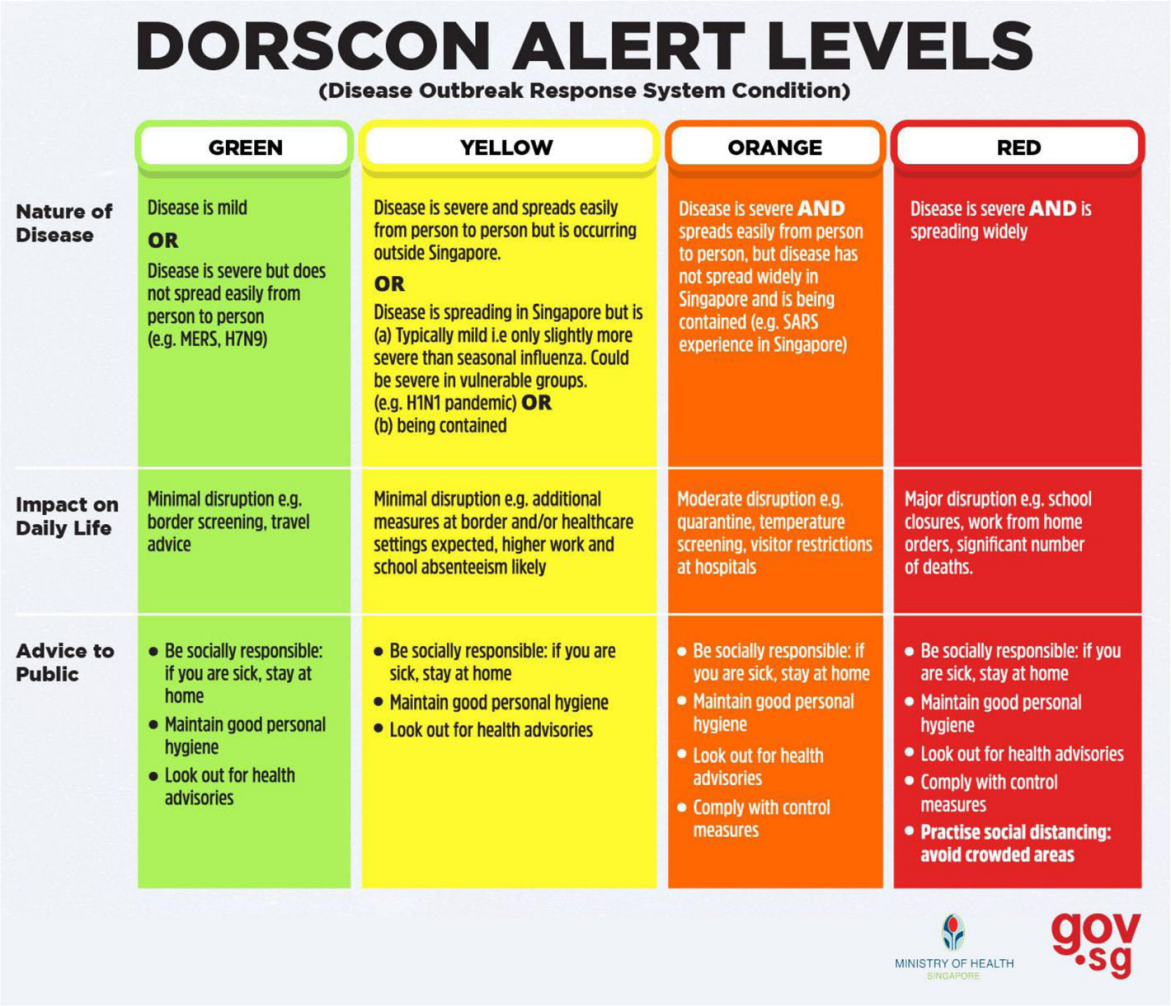
Disease outbreak response system condition by the Ministry of Health (Singapore) [3]

Singapore, one of the earliest countries to be affected by this outbreak, escalated its Disease Outbreak Response System Condition (DORSCON) to Orange status (Fig. 1) on 7 February 2020 in order to put in place measures to control the outbreak: elective surgeries were postponed to reserve hospital beds, staff were segregated into different teams to minimize cross-contamination, and leave cancellation was enforced to ensure sustainability of healthcare services, all these leading to substantial modifications of patient care practices [4, 5].

In our setting of a musculoskeletal department in a tertiary hospital in Singapore, about 500 patients are seen in our outpatient clinics daily, including follow-up visits and new referrals from primary care. Since the outbreak of COVID-19, changes to the workflow were implemented in order to minimize risks of community spread without compromising on non-elective care. Doctors were divided into small teams to maintain small group segregation, whilst administrative staff called individual patients up to reschedule of non-urgent outpatient appointments, with a small group also re-deployed to screening stations in the hospital. Similar measures were also implemented by fellow orthopaedic colleagues combating the COVID-19 pandemic in Italy [6] in order to sustain emergency surgical capabilities and needs including patients with fragility hip fractures [7].

Studies in China assessed the mental health of first-line COVID-19 medical staff using various scales and concluded that the incidence of anxiety and stress is high and care should be provided to this subgroup of healthcare workers [8, 9]. However, we question if significant stress levels might also be observed in medical staff working in subspecialty outpatient departments, who are not first-line care providers of COVID-19 patients. We hypothesize that these healthcare workers also face significant psychological strain, and we aim to objectively determine the prevalence using a validated caregiver strain index.

## Methods

### Participants

We conducted a cross-sectional study between 2 March 2020 and 4 March 2020, a month into the DORSCON Orange status, in a tertiary hospital in Singapore. All frontline healthcare workers working in outpatient musculoskeletal clinics in a tertiary hospital in Singapore were invited to complete this anonymized survey on a voluntary basis. Healthcare workers based in these clinics include doctors, nurses, patient service associates, healthcare assistants, and orthopaedic technicians. The survey was conducted in English, and despite being from different races, all participants spoke English as their first language. Institutional review board exemption was obtained (REF 2020/2661) for this study.

### Data collection

We collected basic demographic data including age, gender, race, marital status, and living status. Vocational status, work experience (number of years at their current job), and average working hours per day were also collected to investigate differences in various roles in a work environment. In order to measure psychological strain in these healthcare workers, we used a 13-question tool adapted from the validated Caregiver Strain Index (CSI). It assesses five main domains including employment, financial, physical, social, and time. Each positive response is awarded a point, and an individual who scores seven points or more indicates a greater level of strain. This index has a high internal consistency reliability (alpha = 0.86) [10, 11].

### Statistical analysis

Based on CSI scores, the participants were divided into 2 groups. Group 1 contains participants who score seven or more, indicating a greater level of strain. Group 2 contains participants who have scored lower than 7 points, indicating a lower level of strain.

The Pearson chi-square test was used to compare the distribution of gender, race, marital status, living status, and vocational status between the two groups. We performed the 2-sample *t* test to determine if there was a statistical significance in the difference between the two groups with respect to age, work experience, and average daily working hours.

All statistical analyses were performed with the SPSS 25.0 (SPSS Inc., Chicago, IL, USA) software, and statistical significance was defined as *p* < 0.05.

## Results

A total of 62 healthcare workers volunteered for this study. The demographic data of the study population is illustrated in Table 1. The average age of the population was 40 years (range, 21–65; SD, 13) with 45 (72.6%) female participants and with the majority race being Chinese (33.9%). Other races include Malay, Indian, Sikh, Javanese, Boyanese, and Pakistani. 62.9% of the study population were married with 96.8% living with family. Most participants (45.2%) were patient service associates, with the remaining made up of healthcare assistants (6.5%), nurses (21.0%), technicians (4.8%), and doctors (22.6%). The average number of years in their current vocation was 10.7 years (range, 0.01–45; SD, 10.4), and they work an average of 8.5 h (range, 4–14; SD, 1.47) per day.

**Table 1:** Population Demographics

		Total (*n* = 62)	Group 1 (*n* = 32)	Group 2 (*n* = 30)	*p*-value
Age (years)		40.0 ± 12.6	40.4 ± 13.4	39.4 ± 11.9	0.756
Gender (F:M)		45 : 17	24 : 8	21 : 9	0.659
Race	Chinese	21	14	7	0.172
	Malay	20	11	9	
	Indian	12	5	7	
	Others	9	2	7	
Marital status	Single	20	11	9	0.762
	Married	39	20	19	
	Widowed	1	0	1	
	Divorced	2	1	1	
Living status	With family	60	32	28	0.138
	With partner	2	0	2	
Vocation	PSA	28	19	9	0.095
	HCA	4	2	2	
	Nurse	13	3	10	
	OT	3	2	1	
	Doctor	14	6	8	
Working hours (hour/day)		8.5 ± 1.5	8.4 ± 1.8	8.6 ± 1.1	0.733
Work experience (years)		10.7 ± 10.4	10.0 ± 9.5	11.4 ± 11.5	0.623

*PSA* Patient service associate, *HCA* Healthcare assistant, *OT* Orthopaedic technician

After tabulation of the final CSI scores, there were 32 participants (51.6%) who had 7 or more positive responses (group 1), and the remaining 30 participants (48.4%) were allocated to group 2. There were no significant differences between the two groups in terms of age, gender, race, marital status, and living status. Vocational status, work experience, and average working hours per day were also not significantly different between both groups.

Amongst the 13 items in the questionnaire (Table 2), “work adjustments” (74.2%), “changes in personal plans” (72.6%), and finding it “confining” (72.6%) garnered the most positive responses in the questionnaire. On the other hand, “financial concerns” garnered the least positive responses (21.0%).

**Table 2:** Thirteen-question tool adapted from Caregiver Strain Index (CSI)

	No. of positive responses in:		
	Total	Group 1	Group 2
1. Sleep is disturbed	18	14	4
2. Work has been inconvenient	37	30	7
3. It is a physical strain (e.g. donning gown and N95 masks, avoid wearing uniform in public)	33	26	7
4. It is confining (e.g. restricts free time, turned down social gatherings)	45	31	15
5. There have been family adjustments (e.g. reduced meetups, self-isolation)	41	28	13
6. There have been changes in personal plans (e.g. unable to go on leave or vacation, had to turn down another job)	45	30	15
7. There have been other demands on my time (e.g. caring for family members)	26	23	3
8. There have been emotional adjustments	29	25	4
9. Some patients’ behavior is upsetting (e.g. facing stigma)	44	28	16
10. It is upsetting to find that patients’ behavior has changed so much from before	37	24	13
11. There have been work adjustments (e.g. change in job scope, reduced interaction with colleagues)	46	31	15
12. It is a financial strain to me (e.g. accommodation fees incurred from self-isolation)	13	12	1
13. Feeling completely overwhelmed (e.g. because I worry about something or concerns about how I will manage)	26	22	4

## Discussion

The global outbreak of COVID-19 has undoubtedly placed tremendous pressure on healthcare resources: retired doctors re-joined the workforce and nursing students were fast-tracked to graduation in order to boost the workforce in Italy, anxiety levels rising amongst US healthcare workers, and a Chinese healthcare system that was overworked and overwhelmed even in peace times and subsequently struggled with this outbreak.

The protracted outbreak has also, less expectedly, transformed the workings of a musculoskeletal department in Singapore. In the operating theatres, only surgeries pertaining to musculoskeletal tumour, trauma, and spinal cord compression were allowed to continue as scheduled. Elective surgeries such as joint arthroplasty and arthroscopies were postponed to later dates in order to ensure adequate beds are available to cope with rising admissions related to the COVID-19 pandemic. In the inpatient wards, teams were designated different sectors of clinical areas to minimize cross-contamination, and referrals from other departments were reviewed by a designated mobile team of doctors who were equipped with personal protective equipment (PPE) when the need arises. This was to ensure that rapid contact tracing and isolation could be performed if there was inadvertent exposure to a confirmed COVID-19 case. In outpatient clinics, patients were screened for risk factors and had their temperature checked with a thermal scanner, with any suspected cases either re-directed to the emergency department or reviewed in an isolation room with full PPE donned. Remote extensions of medical leave as well as home delivery of medications were used to encourage patients to defer any non-urgent appointments [5, 12].

In Singapore, healthcare professionals were required to cancel vacation plans and a portion were re-deployed to the Emergency Department and Infectious Disease centres to cope with overwhelming numbers. Others adopted measures such as self-imposed isolation at a hotel or rental apartments so as to minimize any possibility of transmission to their own family. Extrapolating from the events during the 2003 severe acute respiratory syndrome (SARS) outbreak, we will expect healthcare workers to experience more psychological stress and anxiety symptoms as the outbreak persists [13–15]. China, the first country that managed to “flatten the curve” in this COVID-19 outbreak, started publishing studies about the psychological symptoms faced by frontline COVID-19 healthcare workers only months after the index case [2, 8, 9]. To our knowledge, this is the first study which uses a validated scoring system to assess the strain faced by healthcare workers in an outpatient musculoskeletal clinic setting in the early phase of the COVID-19 pandemic.

The main finding from this study is that increased stressors are not only found in healthcare personnel working in intensive care units or the emergency department as more than half (51.6%) of our study population from an outpatient musculoskeletal clinic were found to have a significant level of strain, a percentage comparable to that of caregivers who provide care for hip fracture patients locally [16] and, more notably, to figures at the conclusion of the SARS epidemic [17]. This could be attributed to the increase in anxiety levels in the general public in response to the COVID-19 outbreak, and healthcare workers bear the brunt of the resulting irrational behaviour. Further, lapses may occur when primary healthcare and emergency departments are overwhelmed, resulting in inappropriate referrals to specialist clinics where patients had coryzal symptoms but are inadequately screened. This could result in an increase in perceived risks of contracting COVID-19.

In addition, our study found no significant difference in the demographics between participants in both groups, illustrating that such an outbreak affects every unit in the healthcare industry regardless of age, gender, race, vocation, and working experience. This could be in part contributed by fast-changing policies and guidelines during a period of adaptation where uncertainty becomes an additional stressor [14]. The prevalence of increased stressors in our study population is comparable to the prevalence of psychiatric symptoms reported in Hong Kong-based healthcare workers during the 2003 SARS outbreak (41–65%) [18–20]. To this date, there are no studies in other countries or regions that investigated the psychological stress faced by healthcare workers in an outpatient specialist clinic setting in response to the COVID-19 global outbreak.

A large majority of the study population quoted adjustments at work as a problem, and this may be attributed to redeployment of manpower to unfamiliar job scopes and requiring remaining staff to be on longer or extra shifts [13]. Another reason could be the frustrations experienced by patients due to postponement of elective clinic appointments and surgeries, which result in displeasure and disagreements. The second most quoted factor in increasing stress was the changes in personal plans healthcare workers had to adapt to during the COVID-19 pandemic. Approved leaves and travel plans had to be cancelled whilst inconvenience and financial costs were incurred when these medical staff had to arrange for alternative accommodation to isolate themselves from family members. These factors should be thoroughly investigated, and the solutions to these problems should be incorporated into a protocol for us to be better prepared for future outbreaks.

One limitation of this study is the small sample size of the study population which limits power analysis. However, this was the full complement of staff in our outpatient department, and further, a larger study over a long time may prevent an accurate depiction of such an acute event. Another limitation is that only one assessment is performed at 1 month after alert levels were raised by the Ministry of Health (Singapore). The lack of assessment prior to the event prevents us from gauging the baseline CSI of the study population. Lastly, the psychological impact of the COVID-19 pandemic may differ amongst healthcare givers working in different departments of the hospital, and our study population may not be representative of all other healthcare providers. Future studies can be conducted to hold more in-depth interviews, as well as to follow up the group of participants to determine the long-term effects of the COVID-19 outbreak, but such interviews are difficult to conduct in the current situation with restrictions on person-to-person contact.

Our survey highlights the fact that increased stress can potentially be experienced by healthcare workers during the COVID-19 outbreak and must not be overlooked. Even in the early phase of the pandemic, caregiver strain was increased in healthcare workers. With the massive rise in number of COVID-19 cases, the psychological strain is likely to affect larger numbers, and perhaps, to a higher extent. Authorities, health agencies, and institutions may need to look into factors contributing to stressors to minimize the unintended consequences this imposes on our healthcare personnel, our precious resource in the fight against this global epidemic. Reasonable support resources should also be made available to these individuals, and stress levels should be monitored. The current most efficient method will be to model after measures adopted by Chinese hospitals such as providing alternative accommodation and food and living supplies for staff to temporarily isolate themselves, developing clear and stringent rules on the use of PPE to reduce confusion and worry, and providing psychological counselling services to provide support whenever needed [2].

## Conclusion

With reports suggesting that the COVID-19 outbreak will likely take on a prolonged course, the mental health of healthcare workers will be the next resource to be exhausted. In the context of an early and controlled disease outbreak in Singapore where health services are not operating past its maximum capacity, a large portion of participants in our study, who are not based in emergency departments or infectious disease units, have already started to experience a greater level of strain. The protracted duration of the COVID-19 outbreak and its resultant prolonged adjustments can have unintended consequences of wearing down healthcare resources otherwise allocated to chronic and elective conditions. Countries should ensure that measures are put in place to safeguard the mental well-being of our healthcare workers to avoid needing another reactive strategy in this battle against COVID-19.

## Data Availability

The datasets used and/or analysed during the current study are available from the corresponding author on reasonable request.

## Abbreviations

COVID-19: Coronavirus disease-19
DORSCON: Disease Outbreak Response System Condition
CSI: Caregiver Strain Index
PPE: Personal protective equipment
SARS: Severe acute respiratory syndrome
